# Subdomain Location of Mutations in Cardiac Actin Correlate with Type of Functional Change

**DOI:** 10.1371/journal.pone.0036821

**Published:** 2012-05-08

**Authors:** Maureen M. Mundia, Ryan W. Demers, Melissa L. Chow, Alexandru A. Perieteanu, John F. Dawson

**Affiliations:** Department of Molecular and Cellular Biology, University of Guelph, Guelph, Ontario, Canada; Cardiovascular Research Institute Maastricht, Maastricht University, The Netherlands

## Abstract

Determining the molecular mechanisms that lead to the development of heart failure will help us gain better insight into the most costly health problem in the Western world. To understand the roles that the actin protein plays in the development of heart failure, we have taken a systematic approach toward characterizing human cardiac actin mutants that have been associated with either hypertrophic or dilated cardiomyopathy. Seven known cardiac actin mutants were expressed in a baculovirus system, and their intrinsic properties were studied. In general, the changes to the properties of the actin proteins themselves were subtle. The R312H variant exhibited reduced stability, with a *T*_m_ of 53.6°C compared to 56.8°C for WT actin, accompanied with increased polymerization critical concentration and Pi release rate, and a marked increase in nucleotide release rates. Substitution of methionine for leucine at amino acid 305 showed no impact on the stability, nucleotide release rates, or DNase-I inhibition ability of the actin monomer; however, during polymerization, a 2-fold increase in Pi release was observed. Increases to both the *T*_m_ and DNase-I inhibition activity suggested interactions between E99K actin molecules under monomer-promoting conditions. Y166C actin had a higher critical concentration resulting in a lower Pi release rate due to reduced filament-forming potential. The locations of mutations on the ACTC protein correlated with the molecular effects; in general, mutations in subdomain 3 affected the stability of the ACTC protein or affect the polymerization of actin filaments, while mutations in subdomains 1 and 4 more likely affect protein-protein interactions.

## Introduction

Heart failure is a major health problem among developed nations and has been described as the emerging health epidemic of the 21^st^ century [1]. Changes in protein interactions in cardiomyofibre sarcomeres lead to abnormal heart function, contributing to the development of heart diseases such as hypertrophic cardiomyopathy (HCM) or dilated cardiomyopathy (DCM) [2]. The understanding of the molecular mechanisms involved in HCM and DCM development have grown beyond early hypotheses that defective actomyosin force generation causes HCM and altered force transmission leads to DCM [3]. We believe that altered interactions between sarcomere proteins lead to disease states through two related mechanisms: intrinsic instability of the encoded protein, resulting in a reduced level of active protein in the heart, or specific protein interaction changes between the altered protein and binding partners.

**Figure 1:**
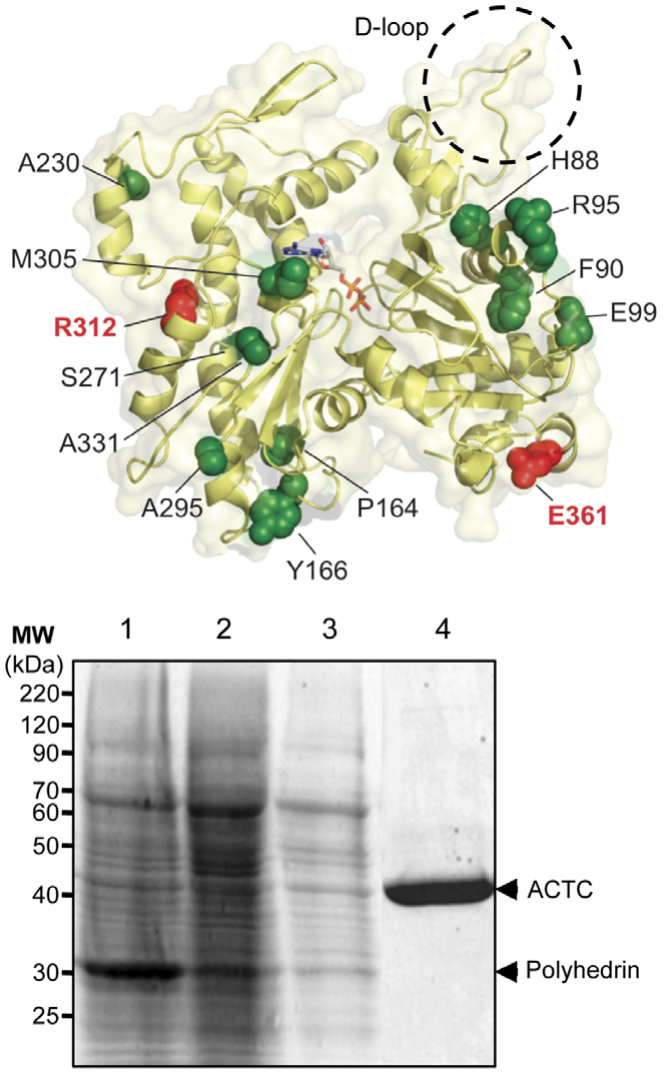
Cardiac actin mutations related to cardiomyopathies. ***A*** *.* The protein structure of actin (PDB 1J6Z) [39] showing the locations of mutations related to HCM (in green spacefilling) or DCM (in red spacefilling) and bound ATP (sticks) visualized using PyMol [40]. The D-loop of actin is highlighted with a dashed circle. ACTC substitution mutations related to HCM shown are: H88Y, F90del, R95C, E99K, P164A, Y166C, A230V, S271F, A295S, R312C, A331P, and M305L. The two ACTC mutants associated with DCM are R312H and E361G. The amino acid numbers listed are based on the sequence of the protein after posttranslational processing of the N-terminus, which removes the first two amino acids of the nascent polypeptide. Note that mutations at Arg-312 are associated with both HCM and DCM. ***B*** *.* Purification of baculovirus expressed ACTC mutant protein. *Sf*9 cells were infected with recombinant baculovirus expressing the A230V ACTC mutant protein at an MOI of 1 for 72 hours. Shown is a 10% SDS-PAGE of samples taken throughout the purification: lane 1, crude lysate; lane 2, supernatant fraction of lysates; lane 3, lysate following filtration and applied to a DNase-I affinity column; lane 4, purified A230V ACTC protein eluted from the DNase-I affinity column. Arrows indicate the position of actin and the polyhedrin protein expressed by the baculovirus in *Sf*9 cells.

To test this hypothesis, we are establishing if variants of the fundamental cardiac actin protein (ACTC) exhibit changes in their biophysical properties that may contribute to cardiomyopathy development. Fourteen known mutants of human ACTC are found in patients that suffer from DCM or HCM (**Figure 1** ***A*** [4]–[9]). Given the often late onset of symptoms, changes in actin activity resulting from these mutations are likely subtle or are overcome by compensatory mechanisms like cardiac remodeling. Nevertheless, it is important to understand the molecular deficiencies associated with these naturally occurring mutants of ACTC as a starting point for understanding the mechanisms underlying the development of heart diseases.

Actin is a 42 kDa protein that is made up of 2 domains and held in its native state by a metal-coordinated nucleotide between the domains. Actin monomers self-associate to form filaments (F-actin) that are an integral part of the muscle contractile machinery. Actin inhibits DNase-I exonuclease activity through a surface loop called the DNase-I binding loop (D-loop). During polymerization, this loop inserts into a hydrophobic cleft in a neighbouring actin protomer [10]. Mutations in this hydrophobic cleft affect actin polymerization [11].

The proper folding of the actin protein requires the eukaryotic chaperonin CCT complex [12]. In the absence of the CCT complex, the unfolding of actin is considered an irreversible process, connected with the loss of metal-coordinated nucleotide from the nucleotide binding pocket. Several techniques have been used to assess actin protein unfolding, including loss of activity assays [13], [14], changes in absorbance [15], differential scanning calorimetry [16], and circular dichroism (CD) measurements [17]. In addition to establishing the protein folding and stability of ACTC variants, we measured nucleotide exchange, polymerization and intrinsic F-actin ATPase activities of the ACTC proteins.

Aside from actin polymerization and protein interaction studies, the biophysical properties of most ACTC variant proteins have not been reported. Some mutations of ACTC associated with cardiomyopathy development have been studied with *in vitro* transcription/translation [18], in yeast actin (ACT1) [19], using baculovirus expression[20]–[22], or in transgenic mouse models [23], [24].

While *in vitro* transcription/translation studies suggested reduced native folding for E99K, P164A, M305L and E361G ACTC variants [18], later work showed that purified P164A and E361G ACT1 proteins from yeast polymerized in a manner similar to WT ACT1 protein [19]. *In vitro* protein stability studies using baculovirus-expressed E99K ACTC yielded contradictory results suggesting that E99K ACTC was stable compared to WT ACTC, with respective T_m_ values of 59.8°C and 60.5°C [20]. Purified A331P yeast actin exhibited a lower T_m_ of 51.6°C compared to 60°C for the WT protein [19].

Previous work shows that the R312H mutation affects actin protein stability, resulting in delayed protein folding by the CCT complex [18] and the inability of viable protein to be purified from yeast [19]. On the other hand, R312H ACTC protein could be successfully purified using baculovirus expression [22]. While this protein forms filaments, the properties of purified R312H ACTC protein were not reported. Similarly, the biophysical properties of E99K and E361G ACTC proteins stably-expressed in transgenic mice were not studied. Resolving some of the conflicting results from previous work was part of our motivation for this study.

No other detailed biophysical information exists regarding the effects of the remaining mutations of ACTC on the actin protein, to the best of our knowledge. As a step toward filling the gap in knowledge regarding the ACTC variants found in patients with HCM or DCM, we have determined if seven ACTC variant proteins possess significantly different intrinsic biophysical and polymerization properties than wildtype ACTC protein. The seven ACTC variants were selected based on the chronology of their discovery in human patients; these were seven of the first nine variants to be discovered that were successfully produced with baculovirus in our laboratory. The A331P and R312H ACTC variants showed stability defects, while the M305L ACTC protein exhibited a fast inorganic phosphate release rate during polymerization. The E99K ACTC protein displayed a *T*_m_ much greater than WT ACTC that could be reduced to a level similar to WT by modification with tetramethylrhodamine, suggesting the formation of intermolecular contacts under monomer-promoting conditions. Finally, the Y166C ACTC protein displayed polymerization defects, confirming the hypothesis that this region of actin is involved with F-actin interactions. Based on the locations and effects of the mutations, we concluded that ACTC variations in subdomain 3 of the actin molecule affect intrinsic stability and/or actin polymerization while mutations in subdomains 1 or 4 do not affect intrinsic actin properties and their role in the development of heart disease is more likely a result of altered interactions with other sarcomere proteins.

## Results

There are fourteen variants of human cardiac actin found in patients with DCM or HCM. Determining the molecular deficiencies associated with the naturally occurring mutants of ACTC is an important starting point for understanding the mechanisms of heart disease development. We purified seven of the first ACTC mutants to be discovered using a baculovirus expression system (**Figure 1** ***B***) and characterized their biophysical properties to determine the effect of these mutations on ACTC function (**Table** **1**).

**Table 1:** Summary of the intrinsic properties of ACTC mutant proteins related to the development of cardiomyopathies in humans.

	T_m_	DNase-I	Nucleotide Release		Cc	P_i_ Release
ACTC	(°C)	IC_50_(nM)	Slow Rate (ms^−1^)	Fast Rate (ms^−1^)	(µM)	(s^−1^×10^−2^)
WT	56.8±1.3	13.2	1.12±2.64	17.47±7.40	0.28±0.13	1.24±0.02
E99K	68.8±0.35*	25.7	2.24±2.18	17.73±5.76	1.67±0.43*	1.25±0.02
	56.7±0.23†					
Y166C	54.1±1.9	24.6	1.62±0.61	16.01±4.56	2.67±0.45*	0.38±0.02*
A230V	53.9±1.9	15.3	2.02±0.42	16.25±4.16	2.11±1.23	1.51±0.24
M305L	55.5±1.7	17.0	2.21±1.94	18.23±1.61	2.21±1.78	2.43±0.03*
R312H	53.6±2.0	36.7	8.87±5.52	181.8±140.7**	4.75±0.27*	2.59±0.05*
A331P	57.4±0.23	26.0	3.63±4.21	22.75±9.05	2.95±1.70	0.88±0.06*
E361G	55.6±1.7	16.8	2.06±1.35	18.11±2.97	0.58±0.48	0.66±0.05*

† labeled with tetramethylrhodamine.

* *p*<0.05 by *t*–test.

** *p*<0.15 by *t–test.*

All values are the average of at least three replicates, showing the standard deviation of each.

### Protein Stability

The thermal stability of a protein reflects its ability to be properly folded and remain in a native state. For actin, the association of metal-coordinated nucleotide is correlated with protein stability [13], [15]. We measured the melting temperature of Ca.ATP-bound ACTC variants employing CD at a scan rate of 1°C per minute. The R312H variant possessed the lowest *T*_m_ of the ACTC proteins, supporting findings of instability with this mutation generated using yeast actin [19] and an *in vitro* transcription/translation system [18].

**Figure 2:**
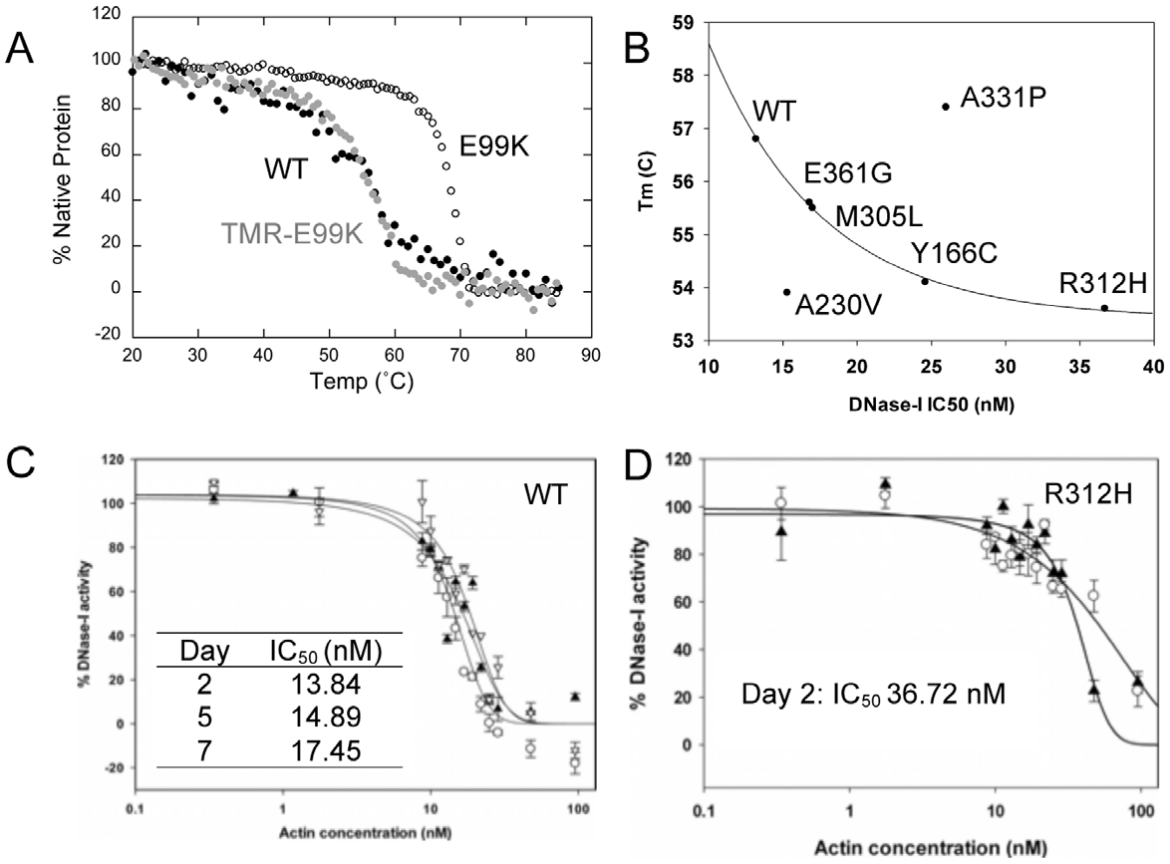
Intrinsic cardiac actin mutant monomer properties. ***A*** *.* The melting temperature of WT ACTC (•) (black circle) was similar to that of TMR-modified E99K ACTC (•) (grey circle) (56.8±1.3°C and 56.7±0.23°C, respectively). Unmodified E99K ACTC (Ο) had a melting temperature of 68.8±0.35°C. ***B*** *.* Correlation of measured *T*_m_ and DNase-I IC_50_ values. The values for five of the tested ACTC proteins fitted a single exponential decay equation (y =  y_0_+A*e* ^-bx^, where y_0_ was 53.4±0.15, A was 19.27±2.65, and b was 0.131±0.01 (S.E.)) with an R^2^ of 0.998). Both ACTC variants with measured *T*_m_ values higher than WT displayed higher DNase-I IC_50_ values (E99K ACTC is not shown), while the A230V ACTC displayed a lower Tm value with an IC_50_ similar to WT ACTC. ***C.*** DNase-I inhibition curves with WT ACTC protein show little change over time (day 2, Ο; day 5, ?; day 7, ▿). Data points are averages of triplicate measurements, with error bars showing standard deviation. ***D.*** DNase-I inhibition by R312H ACTC protein shows a higher initial IC-50 value (day 2, ?). At day 7 (Ο), the IC_50_ data could not be fitted because it had increased.

Interestingly, E99K ACTC protein possessed a significantly higher *T*_m_ of 68.8°C compared to the other ACTC proteins (**Figure 2** ***A***). Since the melting temperature of F-actin is around 67°C [25], we wondered if E99K ACTC might be forming F-actin like contacts even under conditions that are generally known to promote dispersed actin monomers. To test this hypothesis, we modified E99K ACTC with tetramethylrhodamine to inhibit actin polymerization [26], [27]. Using CD, a *T*_m_ for TMR-E99K ACTC of 56.7°C was determined, similar to that of WT ACTC, suggesting that some E99K ACTC self-interactions may occur under conditions that are generally known to promote monomeric actin.

The ability of actin to inhibit DNase-I activity reflects the number of available DNase-I binding sites in a sample of actin [14]. Generally, it is considered that unfolded actin aggregates do not bind DNase-I and therefore, DNase-I inhibition is often used to infer the proportion of folded and unfolded actin within a sample [20]. Other factors such as mutations within the D-loop or actin-actin interactions are also known to impact the ability of the actin protein to inhibit DNAse-I activity [28], [29]. As a result, DNase-I inhibition measures the proportion of monomeric folded actin in a sample and the effect of mutation on DNase-I binding.

If a mutation on actin does not affect the binding of DNase-I, there should be a correlation between DNase-I IC_50_ and actin *T*_m_. When we plotted *T*_m_ values of the ACTC variants against measured DNase-I IC_50_ values, five of the ACTC proteins, including WT ACTC, fell on a fitted single exponential decay curve (**Figure 2** ***B***), suggesting that the amino acid substitutions in these ACTC proteins do not affect their interactions with DNase-I. The R312H ACTC mutant protein had the highest DNase-I IC_50_ value measured one day after purification (**Figure** **2** ***D***). After two days, the R312H ACTC IC_50_ values increased, suggesting a higher proportion of unfolded protein. Conversely, WT ACTC protein exhibited a relatively constant DNase-I IC_50_ over the same period, suggesting that it remains unchanged (**Figure 2** ***C***).

Three ACTC variants deviated from the observed correlation between *T*_m_ and DNase-I IC_50_: E99K and A331P ACTC proteins both had *T*_m_ and DNase-I IC_50_ values higher than WT ACTC, while A230V ACTC had a DNase-I IC_50_ value similar to WT ACTC but with a much lower *T*_m_. It is likely that the mutations of these ACTC variants affect the measurements of *T*_m_ and DNase-I IC_50_ disproportionately. For E99K ACTC, the 2-fold increase in the DNase-I IC_50_ of E99K ACTC compared to WT ACTC suggests that half of the D-loops are inaccessible to DNase-I, perhaps as a result of interactions between the actin monomers suggested above. A similar mechanism may account for the 2-fold increase in IC_50_ for A331P ACTC. On the other hand, a mechanism explaining the low *T*_m_ and normal DNase-I IC_50_ for A230V ACTC is not clear, but may involve local structural instability that does not affect the D-loop as a result of the mutation.

### Nucleotide Interactions

Actin proteins bind a molecule of metal-coordinated ATP in their nucleotide-binding pocket. The hydrolysis of ATP occurs at a higher rate in F-actin, followed by the release of inorganic phosphate (Pi). Defects in nucleotide binding or the ability to hydrolyze ATP might contribute to defects in protein stability or the regulation of actin filaments *in vivo*.

**Figure 3:**
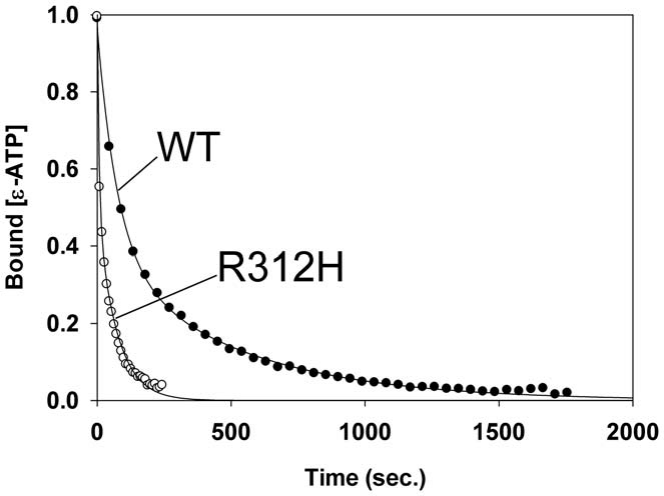
Nucleotide release from monomeric ACTC variant proteins. The release of ε-ATP from WT ACTC followed a 2^nd^-order decay model (•). The R312H ACTC protein showed faster nucleotide release kinetics (Ο) that fitted the 2^nd^-order decay model with a significantly different fast decay rate.

The rate of nucleotide release from all of ACTC proteins tested under monomeric actin conditions fitted a 2^nd^ order decay model (**Figure 3**) with a slow and fast rate occurring. We found no statistical difference between the slow nucleotide release rates for the ACTC mutants. The different decay curves for the R312H ACTC mutant revealed significantly different fast release rates compared to the other ACTC proteins (**Table 1**).

**Figure 4:**
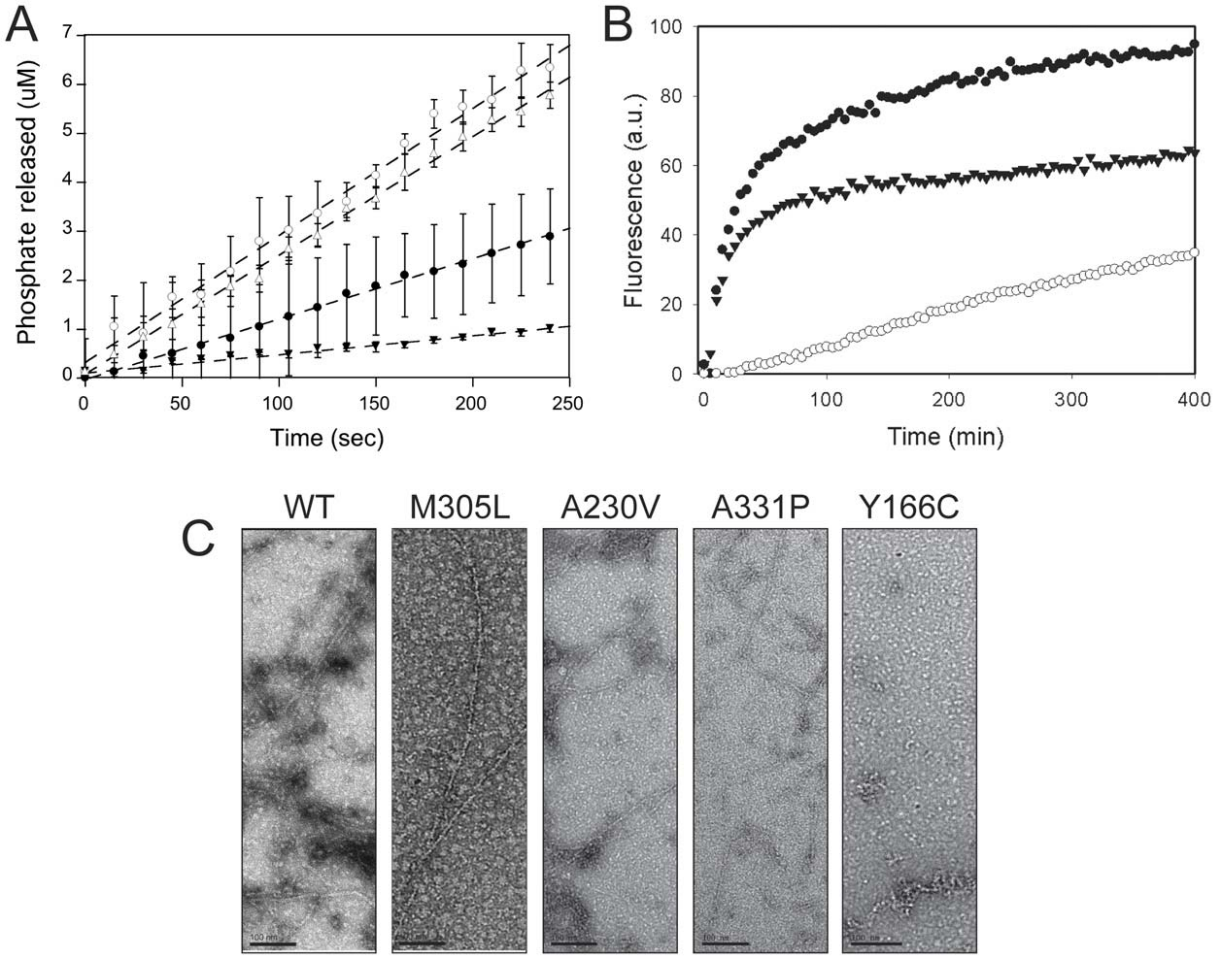
Properties of ACTC mutant proteins under polymerization conditions. ***A*** *.* Polymerization of 10 µM WT (•), R312H (Ο), and Y166C (▾) ACTC proteins in the presence of polymerization salts and 2.5% pyrene-actin. 20% of the data is shown for clarity. ***B*** *.* Phosphate release of WT (•), R312H (Ο), Y166C (▾), and M305L (▵) ACTC proteins under polymerization conditions in the presence of 0.1 µM ATP. Upon the addition of polymerization salts, the release of inorganic phosphate was measured and the total concentration plotted over time. Data points are averages of triplicate measurements, with error bars showing standard deviation and linear regression of the data to determine the specific activity of each ACTC protein. ***C*** *.* Electron microscopy of WT ACTC and mutant proteins, following a 2 hour incubation at room temperature in the presence of polymerization buffer. Note the absence of filaments in the Y166C ACTC protein sample. Images are representative of each ACTC mutant protein shown. Scale bar, 100 nm.

Three classes of Pi release rates emerged from analysis of the ACTC mutant proteins under polymerizing conditions (**Figure 4** ***A***): those similar to WT (E99K and A230V), those with significantly lower Pi release rates (Y166C, E361G, and A331P), and those with significantly higher Pi release rates (M305L and R312H). The R312H ACTC mutant had the highest rate of nucleotide release under non-polymerizing conditions and the highest rate of Pi release under polymerizing conditions. The fast Pi release rate during polymerization of R312H ACTC is likely due to breakage of filaments, resulting in rapid treadmilling and ATP hydrolysis, as seen with other unstable mutant actin proteins [30], [31]. Conversely, the Y166C ACTC protein had the lowest nucleotide release and Pi release rates, suggesting a defect in polymerization.

### Polymerization Properties

To determine the impact of ACTC mutations on the ability of the proteins to polymerize, we measured the critical concentration (Cc) for all of the ACTC variants. We found the R312H, A331P and Y166C ACTC mutants possessed the highest Cc values of the ACTC mutants, reflected in lower maximum F-actin content in polymerization reactions (**Figure 4** ***B***). When ultrastructural details were examined by electron microscopy, we found all of the ACTC mutants formed filaments with normal morphology, except Y166C ACTC. The Y166C mutant ACTC protein did not form regular actin filaments, but rather short filaments and aggregates (**Figure 4** ***C***), confirming a polymerization defect.

## Discussion

At the core of the heart muscle are contractile proteins. We have sought to understand some of the mechanisms of heart failure from the perspective of the cardiac actin protein. While changes to interactions between some ACTC variants and specific proteins have been studied, very little is known about the biophysical properties of naturally occurring ACTC mutants related to heart disease themselves.

When the ACTC mutants were ranked within each assay, two main groups were observed: those ACTC mutants with changes in actin protein stability and those with changes in actin protein polymerization. However, it should be noted that the magnitudes of any changes in these ACTC mutant proteins were subtle. These subtle changes might reflect the late development of some HCM or DCM cases in humans.

We observed a strong correlation between *T*_m_ values and DNase-I IC_50_ values for five of the ACTC proteins examined. However, for E99K and A331P ACTC, intermonomer interactions may bury some of the D-loops of ACTC, resulting in disproportionate changes in the two parameters. This is most likely the case for E99K ACTC, where modification with TMR reduced an elevated *T*_m_ reminiscent of F-actin to that of monomeric WT ACTC. It is not clear if such interactions would impact the heart, since actin filaments form for both E99K and A331P ACTC proteins and a host of regulatory proteins surround the actin filament *in situ* to encourage proper filament structure.

**Figure 5:**
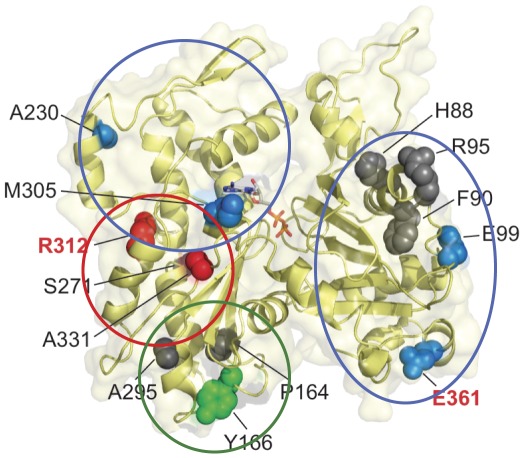
Correlation of location and effects of ACTC mutations. The locations of ACTC mutations associated with cardiomyopathies are shown in black (HCM) and red (DCM) letters. The regions of molecular effects are circled and spacefilling models presented in red (protein stability changes), green (actin polymerization changed), and blue (no significant intrinsic property changes). Those residues shown in grey have not been characterized. Coordinate data for ATP-bound TMR-actin (1J6Z) [39] were visualized using PyMol [40].

Our rankings suggested a pattern where intrinsic deficiencies occur with mutations in subdomain 3 of actin (**Figure 5**, R312H, A331P and Y166C) while mutations in subdomains 1 and 4 do not affect the intrinsic properties of the actin, but more likely affect protein-protein interactions. The Y166C ACTC mutant exhibited polymerization deficiency through an increased Cc, abnormal filament morphology, and lowered P_i_ release. This mutation is located in the hydrophobic cleft of actin which is known to be involved in F-actin contacts [10]. Similarly, studies on yeast actin show that mutations within the W-loop (a.a. 165–172) of yeast actin also impact actin polymerization. [11].

The R312H ACTC mutant possessed reduced protein stability accompanied with higher polymerization Cc, and increased nucleotide and Pi release rates. The fast production of inorganic phosphate during polymerization of R312H ACTC is likely due to breakage of filaments, resulting in rapid treadmilling and ATP hydrolysis, as seen with other unstable mutant actin proteins [30], [31]. These observations are confirmed by previous work showing the effect of the R312H mutation on actin protein stability [18], [19], [32]. Altered contractile protein interactions were observed with reconstituted regulated R312H ACTC filaments leading to increased calcium sensitivity [22], [33]. It is not clear how instability of the R312H ACTC variant is related to changes in protein interactions; however, the inclusion of the filament stabilizing toxin phalloidin to the assays may have influenced the behaviour of the variant ACTC protein.

The A331P ACTC mutant also follows a pattern of altered protein stability, but with less severity. Ala-331 is located in subdomain 3 of actin, beside Pro-332 and Pro-333 in the hinge region between the large and small domains [34]. The addition of another Pro residue results in faster nucleotide release rates, supporting the hypothesis that movement of this region is critical for nucleotide binding [35], [36]. A331P ACTC protein also displayed a relatively high F-actin Cc and a slow inorganic phosphate release rate, suggesting a slight defect in polymerization., something that was seen with A331P yeast actin [19]. The yeast actin A331P protein T_m_ of 51.6°C as measured by CD [19] also agrees with the hypothesis that A331P ACTC is structurally unstable. However, the A331P ACTC protein showed a *T*_m_ higher than WT ACTC protein. Intermonomer interactions similar to those likely occurring E99K ACTC may also occur with A331P ACTC to affect the *T*_m_ and DNase-I IC_50_.

Aside from potential intermolecular interactions of E99K ACTC protein under monomeric conditions, ACTC mutations in subdomain 1 of actin (E99K and E361G) did not otherwise adversely affect the intrinsic properties of the actin molecule itself and more likely affect protein interactions. Previous work has shown that the E99K mutation affects myosin and tropomyosin binding [20], [22], while the E361G mutation affects α-actinin binding [19] and the regulation of actin filaments by troponin I [23].

The locations of both A230V and M305L mutations in subdomain 4 are not thought to be involved in important F-actin contacts or actomyosin interactions. The increase in Pi release rate of M305L ACTC protein may reflect the location of the mutation within the nucleotide-binding cleft [34]. The A230V ACTC mutation is believed to be in the tropomyosin-binding site [37].

Based on the pattern emerging from our data, we predict that the remaining ACTC mutations located in subdomain 3 (P164A, S271F, A295S, and R312C) affect protein stability or actin polymerization, while mutations in subdomain 1 (H88Y, F90del, R95C) impact protein interactions with other sarcomere proteins. We can now examine the interaction of the ACTC proteins with important actin binding proteins, including myosin, thin filament regulatory proteins, and myosin binding protein-C to test our model and further illuminate the molecular alterations that occur with changes in the ACTC protein that are related to the development of cardiomyopathies.

## Materials and Methods

### Reagents

Unless otherwise stated, all reagents were purchased from either Sigma-Aldrich (St. Louis, MO) or Fisher Scientific (Mississauga, ON).

### Recombinant Baculovirus Generation

The E99K-ACTC mutant and WT ACTC recombinant viruses had previously been produced [21]. ACTC point mutants leading to amino acid substitutions R312H, E361G, P164A, A331P, Y166C, A295S, A230V and M305L were produced in the human ACTC cDNA using the one-step Quikchange site-directed mutagenesis system (Stratagene, La Jolla, CA) with oligonucleotides containing *BamHI* restriction sites at the 5′ and 3′ ends and subcloned into the pTOPO2 vector using TOPO-TA cloning (Invitrogen, Carlsbad, CA). The mutant ACTC DNA was subsequently *BamHI* digested out of the TOPO vector and ligated into *BglII* digested pAcUW2Bmod vector. The orientation of the insert, mutagenesis, and absence of other PCR-induced errors were verified by direct DNA sequencing.

Recombinant viruses were produced by co-transfecting *Sf*9 insect cells with the pAcUW2Bmod mutant-*ACTC* constructs and linearized baculovirus DNA in the presence of Bacfectin (Clontech, CA). The presence of the ACTC cDNA in the amplified recombinant viruses was verified by PCR and the viruses were plaque-purified as previously described [21].

### Purification of ACTC Proteins

Approximately 1×10^9^ Sf9 cells infected at a multiplicity of infection (MOI) of 1 with recombinant baculovirus were harvested at 72 h post-infection by centrifugation and the ACTC mutant protein was purified as described [21], with the addition of the cleared cell lysates being passed through glass wool twice to remove residual cell debris and lipids before purification by DNase-I affinity chromatography. Purified actin was concentrated with an Amicon concentrator 10,000 MWCO (Millipore, Billerica, MA) and protein concentration determined with Bio-Rad Protein Assay dye reagent (Bio-Rad, Hercules, CA) employing purified α-skeletal G-actin as a standard. The purity of the protein was checked by SDS-PAGE and then stored at 4°C. Tetramethylrhodamine labeling of E99K ACTC protein on Cys374 was carried out using established methods [15].

### Pyrene-actin Polymerization Assays

The polymerization of each ACTC mutant was assessed with a pyrene-fluorescence-based assay [38]. Briefly, actin samples in G-buffer (2 mM Tris, pH 8.0, 0.2 mM CaCl_2_, 0.2 mM ATP, 0.5 mM βME) were mixed with 2.5% pyrene-labeled α-skeletal actin and the fluorescence monitored before and after addition of polymerization buffer (25 mM Tris, pH 8.0, 50 mM KCl, 1 mM EGTA, 2 mM MgCl_2_ and 0.1 mM ATP final concentration) in a Varian Cary Eclipse fluorometer (Varian Inc, Walnut Creek, CA) with excitation and emission wavelengths set at 347 nm and 407 nm respectively and the slit width at 2.5 nm for the excitation and 5 nm for the emission. The critical concentration was determined as the x-intercept of the linear regression derived from fitting the change in pyrene-fluorescence (y-axis) from each of the polymerization profiles of different actin concentrations (x-axis) using the statistical software SigmaPlot 11 (Systat software Inc, San Jose, CA).

### DNase-I Inhibition

The folding and stability of the purified ACTC proteins were assessed using a high throughput DNase-I inhibition assay as described previously [14]. Briefly, 0 to 100 nM ACTC protein and 15.4 nM DNase-I in G-buffer were incubated in 50 µl reactions for 30 min at room temperature in a UV transparent 96 well microplate (Corning Inc., Corning, NY). The assay was initiated by adding 200 µl of 240 µg/ml salmon sperm DNA prepared in DNA buffer (100 mM Tris pH 8, 4 mM MgCl_2_, 1.8 mM CaCl_2_) in each well of the plate. The absorbance was immediately monitored at 260 nm over a period of 3 min using a UV plate reader (BioTek, Winooski, VT). The percentage of DNase-I activity was determined by comparing the initial linear rates of DNase-I activity in assays containing ACTC to control reactions containing no actin. The IC_50_ of DNase-I by ACTC was determined as the concentration of the ACTC protein that causes 50% inhibition of DNase-I activity. DNase-I activity plots were generated using SigmaPlot 11 (Systat Software, San Jose, CA).

### Thermal Stability

The thermal stability of ACTC samples was determined using circular dichroism (CD) spectrometry. ACTC samples were dialyzed in HEPES G-buffer (2 mM HEPES, pH 8.0, 0.2 mM CaCl_2_, 0.2 mM ATP, and 0.2 mM βME) with at least 3 buffer exchanges. Following dialysis, the concentrations of the actin samples were determined using the Bio-Rad Protein Assay dye reagent (Bio-Rad, Hercules, CA) with purified α-skeletal G-actin as a standard and the proteins were diluted to 0.16 mg/ml in HEPES G-buffer. The negative ellipticity of the actin protein samples was measured at 222 nm with an increasing thermal gradient from 20–85°C at a 1°C/min scan rate using a JASCO J-815 Chiro-optical spectrometer (JASCO, Easton, MD). The rate of change in ellipticity at 222 nm was plotted as a function of temperature, a Weibull fit was applied using SigmaPlot 11 (Systat Software, San Jose, CA), and the minimum was taken to be the melting temperature (*T*_m_), as described previously [15].

### Pi Release

The inorganic phosphate released from actin samples upon the initiation of polymerization was monitored using the EnzChek phosphate assay kit (Invitrogen, Burlington, ON). Reactions (250 µl) containing 10 µM ACTC in G-buffer, 400 µM MESG, and 0.5 U purine nucleoside phosphorylase were incubated for 15 min at room temperature before polymerization to remove any inorganic phosphate present in solution. Following incubation, polymerization salts were added to a final concentration of 50 mM KCl and 2 mM MgCl_2_ to initiate polymerization and the absorbance at 360 nm monitored using a Beckman Coulter DU800 spectrophotometer (Beckman, Mississauga, ON). Measurements in the absence of MESG were baseline-subtracted from the experimental data to account for light scattering of polymerizing actin samples. A standard curve of 0 to 100 µM inorganic phosphate was used to convert absorbance at 360 nm to inorganic phosphate concentration. Phosphate release rates were plotted using SigmaPlot 11 (Systat Software, San Jose, CA).

### Nucleotide Release

The rates of ε-ATP release from ACTC proteins were determined by the change in the fluorescence of ε-ATP-bound ACTC using a stopped-flow fluorometer. Unbound ATP was removed from 10 µM ACTC in G-buffer containing 10 mM Tris-HCl, pH 8.0, using Bio-Rad AG 1-X8 anion exchange resin (Hercules, CA) equilibrated in the same buffer. Following removal of unbound ATP, 0.1 mM ε-ATP was added and incubated on ice at 4°C for 2 h. Unbound ε-ATP was then removed using Bio-Rad AG 1-X8 anion exchange resin (Hercules, CA) equilibrated in G-buffer containing 10 mM Tris-HCl, pH 8.0. 10 µM ε-ATP was then added to the solution and the actin concentration determined using Bio-Rad Protein Assay dye reagent (Bio-Rad, Hercules, CA) employing purified α-skeletal G-actin as a standard. Prior to use in the experiment, ε-ATP-bound ACTC proteins were diluted to 2 µM using G-buffer containing 10 mM Tris-HCl, pH 8.0, and 10 µM ε-ATP.

2 µM ε-ATP-bound ACTC proteins were rapidly mixed with an equal volume of G-buffer containing 10 mM Tris-HCl, pH 8.0, 0.2 mM CaCl_2_, 0.2 mM ATP, and 0.2 mM βME, at 22°C using an Applied Photophysics SX20 stopped-flow fluorometer (Applied Photophysics Ltd., Surrey, UK) with an excitation wavelength of 350 nm, excitation slit width of 4.65 nm, and a 395 nm emission cutoff filter. The assay was performed in at least triplicate for each ACTC-mutant protein per preparation. The ε-ATP fluorescence decay signal for ACTC-mutant proteins was plotted against time and was fitted to a second order decay fit using SigmaPlot 11 (San Jose, CA) to determine the nucleotide release rates.

### Electron Microscopy

Electron micrographs of the ACTC polymerization reactions were taken to examine the morphology of the filaments. 10 µM of the actin proteins were incubated for 2 h at room temperature under polymerization conditions. 3 µl of the polymerization reactions were immobilized on carbon-coated grids and the grid subsequently negatively stained with 2% aqueous uranyl acetate. Filaments were viewed with a Leo 912ab™ electron microscope with an in-column Omega energy filter in the Microscopy Imaging Facility at the University of Guelph.

### Statistical Analysis

The statistical significance of the critical concentration, melting temperature and inorganic phosphate release values for the ACTC mutants compared to WT ACTC were evaluated by a *t-test* (α = 0.05) using GraphPad Software, Inc. (La Jolla, CA). To understand the statistical significance of each ACTC mutant nucleotide release rate, a *Grubb’s* test (at 95% confidence interval) was used to identify outlying data points for each ACTC protein tested. The means of the remaining data points are reported and significant differences compared to WT ACTC were evaluated by a *t-test* (α = 0.15).
